# On the sensitivity of event-related potentials to retrieval mode

**DOI:** 10.1016/j.bandc.2019.103580

**Published:** 2019-10

**Authors:** Angharad N. Williams, Edward L. Wilding

**Affiliations:** a Cardiff University Brain Research Imaging Centre (CUBRIC), School of Psychology, Cardiff University, Cardiff, United Kingdom; b School of Psychology, Nottingham University, Nottingham, United Kingdom

**Keywords:** Retrieval preparation, Retrieval mode, Episodic memory, Task-set, Task-switching, Event-related potentials (ERPs)

## Abstract

Event-related potential (ERP) signatures of preparation to retrieve episodic memories have been identified in several studies. A common finding is relatively more positive-going ERP activity over right-frontal sites when people prepare for episodic rather than semantic retrieval. This activity has been linked to the process of retrieval mode – a retrieval set that ensures subsequent events are treated as cues for episodic retrieval. This experiment was designed to test one explanation for why this putative index of retrieval mode was not observed in two recent experiments. Towards this end, ERPs were recorded time-locked to different task-cues indicating which of two retrieval tasks participants should prepare to complete. Each task-cue was followed by a retrieval-cue that required a memory judgment. Departures from the designs of the two studies in which null ERP results were obtained were intra-trial timings and the order in which task cues were presented. Frequentist statistics revealed that ERPs elicited by the task-cues did index preparation to retrieve. The topographies of these activities, however, did not overlap markedly with that of the putative index of retrieval mode reported previously. Bayesian analyses, moreover, provided little compelling evidence for a signature of retrieval mode. These outcomes prompt consideration of how ERP sensitivities to preparatory retrieval processing should be characterized.

## Highlights

• ERPs were acquired while people prepared to make memory judgments.
• A commonly observed ERP modulation associated with preparation for episodic memory retrieval was not obtained.
• The outcomes suggest that the functional significance of this modulation merits re-consideration.

## 1 Introduction

Tulving (1983) introduced the concept of retrieval mode. He suggested that people enter this mode when preparing to recover episodic memories, and that it can influence memory judgments because it ensures events are treated as cues for episodic retrieval (Wheeler, Stuss, & Tulving, 1997). Candidate brain regions supporting retrieval mode were first identified in position emission tomography (PET) and then in functional magnetic resonance imaging (fMRI) studies of memory retrieval (Kapur et al., 1995, Lepage et al., 2000). This process has also been studied using event-related potentials (ERPs), most frequently in designs where neural activity has been measured time-locked to task-cues indicating which kinds of memory judgments people should prepare to make. A common finding is that this preparatory activity varies when people prepare to complete episodic compared to semantic retrieval tasks (Wilding & Herron, 2006). The difference takes the form of a temporally extended positivity at right-frontal scalp locations when people prepare for episodic rather than semantic retrieval (Düzel et al., 1999, Düzel et al., 2001, Herron and Wilding, 2004, Herron and Wilding, 2006a, Herron and Wilding, 2006b, Morcom and Rugg, 2002).

Düzel et al., 1999, Düzel et al., 2001 were the first to link this pattern of ERP activity to retrieval mode, and the case for this link has been strengthened in subsequent work, where similar ERP modulations have been observed when different episodic demands have been imposed (Morcom & Rugg, 2002). These data points are consistent with Tulving’s proposal (Tulving, 1983) that retrieval mode is engaged whenever any form of episodic retrieval is required (for a direct comparison between ERPs elicited in different episodic tasks, see: Herron & Wilding, 2004).

The experiments in which this right-frontal ERP effect has been observed have often included requirements to switch frequently between two tasks, and neural and behavioral measures have been assessed for switch and for repeat trials. Switch trials are those where the preparatory cue is different on the preceding trial. Repeat trials are those where the cue on the preceding trial matches that for the trial at hand (Monsell, 2003b). This separation has been employed because it provides an opportunity to align neural measures linked to preparation to retrieve with the consequences of doing so, under the assumption that the opportunity to engage fully in retrieval mode will be greater on repeat than on switch trials (Morcom & Rugg, 2002).

Changes in performance across switch and repeat trials – switch costs – have been documented extensively across many different kinds of task (for a review, see: Vandierendonck, Liefooghe, & Verbruggen, 2010). A common finding is slower reaction times on switch than on repeat trials, and this outcome has been replicated in experiments where people switch to and from a task requiring episodic retrieval (Wilding and Herron, 2006, Williams et al., 2016). The most common ERP finding in these memory tasks is that right-frontally distributed activity associated with retrieval mode is evident on repeat trials only (Wilding & Ranganath, 2012). This outcome is compatible with the view that the neural activity on repeat trials indexes successful preparation to retrieve and plays a role in the performance change on repeat relative to switch trials (Herron & Wilding, 2006a).

The starting point for the work here is two experiments in which there was no evidence for the right-frontally distributed signature described above (Williams et al., 2016). In the first of these, participants initially saw pictures of objects located either inside or outside a picture of a building frame. In a subsequent test phase a task-cue on each trial indicated whether they should prepare to make an episodic judgment: remember location (inside/outside), or a semantic judgment: ‘where is this object typically found (inside/outside)?’ The task-cue sequence was predictable and a switch occurred every other trial. ERPs were acquired time-locked to the task-cues, and the only change in the design of the second experiment was the use of a different semantic task. This required a judgment about the typical size of the object that was shown.

In both experiments the time taken to make decisions was shorter on repeat trials, replicating the modal finding and indicating that participants engaged with the task demands. In the first experiment, reliable temporally extended differences elicited in response to the task-cues were revealed on both switch and repeat trials. For both trial-types the largest differences consisted of more positive-going activity elicited by the semantic than by the episodic task-cues from 800 to 1900 ms post-stimulus. For switch trials these differences were largest at right-frontal locations while for repeat trials the divergences were most prominent at the vertex. In the second experiment differences between the ERPs elicited by the different task cues only approached significance. In addition to these frequentist outcomes, Bayesian analyses were conducted (Williams et al., 2016). These were restricted to right-frontal electrode locations and comprised assessments of the critical ERP data as a replication of the effects obtained in a prior study (Evans, Williams, & Wilding, 2015). In both cases there was substantial evidence for the view that the previously reported right-frontally distributed modulation had not been replicated.

Williams et al. (2016) noted that these data are consistent with the view that the right-frontally distributed signature identified in previous studies is not an index of retrieval mode: if it were then it should be revealed when there is a requirement for episodic retrieval, irrespective of the type of episodic retrieval that is required (Düzel et al., 1999, Tulving, 1983, Wheeler et al., 1997). In addition, there are other data points that are consistent with this view. Wilckens, Tremel, Wolk, and Wheeler (2011) required people to switch between making recognition memory and semantic memory judgments to pictures of nameable objects. Neural activity varied reliably in response to task-cues on repeat trials only. This differentiation was most prominent at the vertex, where it was more positive-going for the semantic rather than the episodic task. Wilckens et al. (2011) employed an average reference when analyzing and presenting their data, while Williams et al. (2016) employed the average of the signal at the left and right mastoids. The contrast across the studies is therefore imprecise, although often differences between scalp distributions for these two reference options are not extensive (cf. Curran, 2000, Dien, 1998).

Given the number of experiments in which indices of mode have been reported, Williams et al. (2016) also considered whether elements of their experiment design reduced their likelihood of observing a signature of retrieval mode. One possibility is that the null outcomes they reported arose because participants learned the task-cue sequence. This is feasible given that the sequence was predictable: it changed every third trial (for similar considerations in other contexts, see: Monsell, 2003a, Rogers and Monsell, 1995). If participants learned the sequence, then they might start to prepare for the next trial immediately or soon after making a memory judgment on the previous one. A consequence of this would be a reduction in the magnitude of any ERP signature reflecting preparation to retrieve. This is because the activity of interest would have started before presentation of the task-cue relative to which the activity of interest was measured.

The argument for consideration of the predictability of task-cue sequences in the context of the key null outcomes is given added weight by the fact that unpredictable task-cue sequences have been employed in the majority of experiments in which neural activity indexing preparation to retrieve has been observed. In one notable exception, however, Evans et al. (2015) employed a predictable task-cue sequence and observed an ERP modulation on switch trials that had temporal and spatial characteristics similar to that ascribed to retrieval mode. What may be important in this case is the fact that the experiment timing they employed differed from that in the two experiments due to Williams et al., 2016. Evans et al. (2015) had only a 500 ms (ms) interval between the response on trial *n* and the presentation of the task-cue on trial *n* + 1. This short response-cue interval (RCI) differs from the 1200 ms RCI in the two studies reported by Williams et al. (2016).

How might this timing difference between the experiment designs be important? In both studies there is the possibility that participants learned the task-cue sequence and started to prepare for the next trial in advance of the next task-cue. The extent of that preparation, however, is presumably more limited with a 500 ms RCI than with a 1200 ms RCI. If this is the case, then shorter RCIs will increase the likelihood of observing indices of preparation to retrieve when these are measured time-locked to a subsequent task-cue.

This possibility still has the challenge of explaining why there were some divergences between the ERPs elicited by task cues in the first experiment Williams et al. (2016) reported. None the less, we would argue that the foregoing discussion provides two pointers to ways in which the designs of the experiments reported by Williams et al. (2016) might be changed in order to maximize the likelihood of observing the putative index of retrieval mode. First, the task-cue sequence should be unpredictable, because participants are unlikely to engage in task-specific preparation if the upcoming task is not known. Second, the RCI should be short, thereby limiting the extent of preparation for the upcoming task before the relevant task-cue is shown. Following this reasoning, in the experiment described below we employed the stimuli used previously by Williams et al. (2016) and we made two design changes. First, the task-cue sequence was unpredictable. Second, the RCI was reduced to 500 ms.

At issue is the sensitivity of ERPs to processes that are engaged when people prepare to retrieve information from episodic memory. Failure to observe the putative ERP correlate of retrieval mode in this experiment would encourage re-consideration of its functional significance and prompt wider consideration of how best to characterize the array of processes that are in play when preparation for episodic retrieval is underway.

## 2 Method

### 2.1 Participants

Data from 32 participants (mean age = 23, range = 18–30, 23 female) were included. Data from a further three were excluded due to excessive EEG artefacts. The minimum sample size was based on power analyses for replication attempts of previous outcomes (Evans et al., 2015, Herron and Wilding, 2004, Herron and Wilding, 2006a) (average effect size: *d_z_* = 0.57, α = 0.05, 1 − β = 0.80, N = 22) and set at 24 to accommodate within experiment counterbalancing. A maximum sample size of 32 was again guided by power analyses: this N exceeds the largest sample size (N = 30) given from power analysis of the smallest previous effect size (*d_z_* = 0.47, main effect of cue-type: encoding operation versus semantic task Herron & Wilding, 2004). The final sample size employed was guided via the Bayesian Stopping Rule (Dienes, 2011) which was applied to the Bayesian analyses with a first look conducted at 24 participants, and in this case data collection was terminated at the pre-determined maximum of 32 (cf. Campbell, Chambers, Allen, Hedge, & Sumner, 2017). All participants were right-handed, and reported normal or corrected-to-normal vision. None of the participants had a diagnosis of dyslexia, and they were all native English speakers. At the time of testing no participants reported using psychotropic medication. Participants gave written informed consent before participating, and were paid £10 per hour with each testing session lasting no more than two hours. Cardiff University School of Psychology Ethics Committee reviewed and approved this research.

### 2.2 Design

Stimuli were 240 black line drawings of objects, selected from the International Picture Naming Project Database (Szekely et al., 2004). The corresponding name for each object was between three and ten letters in length, the percentage picture naming frequency was above 0.80 in all cases, and the frequency range was between zero and 7.40 CELEX log transformed. The objects were presented on a monitor with a white background, positioned one meter directly in front of participants. At study, the stimuli subtended maximum visual angles of 5.4° vertically and 8.5° horizontally. At test, objects were presented in the center of the screen subtending maximum visual angles of 1.6° vertically and 1.7° horizontally.

The objects were classified into one of three semantic categories, according to where they were commonly found: inside, outside or both. An object was classified as ‘inside’ if it was usually found inside, and it was classified as ‘outside’ if it was usually found outside. An object was classified as ‘both’ if it could commonly be found both inside and outside. There were 80 objects in each semantic category, and for this classification the mean inter-rater reliability of three raters was 0.72. The experiment had five study-test cycles, and the 80 stimuli from each semantic category were assigned randomly to one of five lists. Thus, each list contained 48 objects with 16 from each semantic category (inside/outside/both). Two additional practice blocks, half the length of the other five study-test blocks, were formed and used before the task proper to familiarize participants with the experiment demands.

**Fig. 1:**
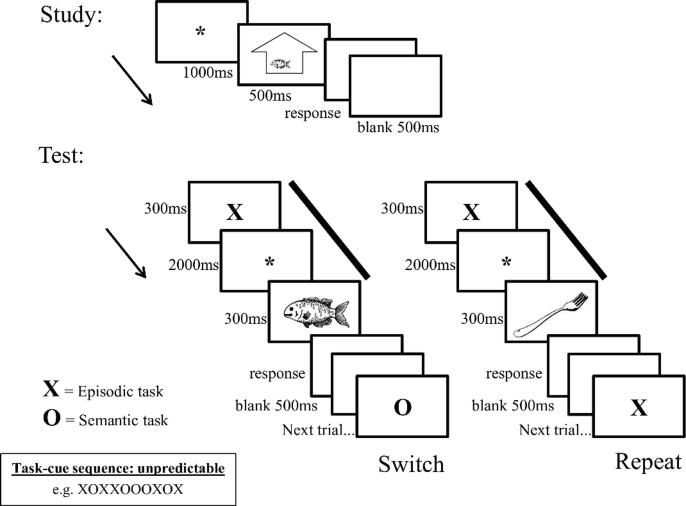
Adapted from Fig. 1 (Williams et al., 2016). A schematic illustration of trial sequences and timing at study (upper panel) and on switch and repeat trials at test (lower panel). The preparatory period of interest for assessing ERPs is indicated by the solid bars.

At study, 24 of the objects were either presented inside or outside an abstract outline of a building (see Fig. 1). They were displayed in one of eight randomized locations (four inside, four outside). Half of the objects were presented inside and half of the objects were presented outside, and this was counterbalanced across individuals. Participants were asked to indicate whether the object appeared inside or outside, and to make a response via button press with their middle or index fingers, respectively. The hand used was counterbalanced across participants.

At test, the 24 objects from the preceding study phase were randomly intermixed with 24 unstudied objects. Each test object was preceded by one of two task-cues presented in the center of the screen. These cues indicated which task participants were to complete. An ‘X’ directed participants to prepare for the episodic task, where they were required to retrieve the prior study location of the object. An ‘O’ directed participants to prepare for the semantic task. This task required identification of the common location of the object depicted, regardless of the study phase. Each test block (for each task) was constructed such that it contained one four-trial run, two three-trial runs, five two-trial runs, and four one-trial runs. The order of these was randomized within each test block. This meant that within each block there were 12 switch trials and 12 repeat trials for each task. The data included in the analyses described below are for the first repeat only, for which there were 8 in each block.

Whether an object appeared on a switch or repeat trial, its task status (episodic/semantic), and old/new status, were counterbalanced across participants. During the test phase, a three-way response was required in each task: episodic task: old-inside/old-outside/new, semantic: inside/outside/both. The inside/outside responses were made using the same fingers as at study, with the addition of the index finger of the other hand to indicate ‘new’ or ‘both’, for the episodic and the semantic task, respectively.

### 2.3 Procedure

At study, a fixation asterisk was presented for 1000 ms, followed by an object (presented inside or outside the building frame outline) for 500 ms. The monitor was then blank until a response was made, and remained blank for 500 ms after each response before the next trial began.

At test, the task-cue (‘X’ or ‘O’) appeared for 300 ms, followed by a fixation asterisk for 2000 ms. Following the ‘X’ cue, participants were to prepare to retrieve information about whether the object appeared inside or outside at study, or whether the object was new (response: inside/outside/new). Following the ‘O’ cue, participants were to prepare to identify where the object was most commonly found: inside or outside, or both inside and outside (response: inside/outside/both). An object was then presented in the center of the screen for 300 ms. The monitor was then blank until a response was made, and remained blank for a further 500 ms (the response cue-interval: RCI) before the next trial began (Fig. 1).

Participants were asked to respond as quickly and as accurately as possible. As in previous work (Williams et al., 2016), trials on which responses were faster than 300 ms or slower than 4000 ms were counted as errors and excluded from the analyses (0.9% of the trials overall).

### 2.4 Electroencephalogram (EEG) procedures

EEG was recorded from 25 silver/silver chloride electrodes housed in an elasticized cap. The arrangement of these was based on the international 10–20 system (Jasper, 1958), encompassing midline (Fz, Cz, Pz) and left/right hemisphere locations for fronto-polar (Fp1/Fp2), frontal (F7/F8, F5/F6, F3/F4), central (T7/T8, C5/C6, C3/C4), posterior (P7/P8, P5/P6, P3/P4), and occipital (O1/O2) sites. Additional electrodes were placed on the left and right mastoid processes, and electrooculogram (EOG) measures were recorded from additional bipolar electrodes placed above and below the right eye (vertical, V-EOG) and on the outer canthi (horizontal, H-EOG). EEG was acquired relative to an average reference with a bandwidth of 0.03–40 Hz (24 dB/oct) and sampled continuously at a rate of 4 ms per point (250 Hz). Impedance at each electrode/scalp interface was below 5 KΩ at the start of each recording session. The data were re-referenced off-line to the average of the signal at the two mastoids. Trials containing large EOG artefact were rejected, as were trials containing A/D saturation or baseline drift exceeding ±75 µV. Other EOG blink artefacts were corrected using a linear regression estimate (Gratton, Coles, & Donchin, 1983). ERPs were segmented time-locked to the preparatory cues, with an epoch length of 2500 ms including a 200 ms pre-stimulus baseline relative to which all mean amplitude measures were computed.

The first trial in each test block was removed from analyses, as it is neither a switch nor a repeat trial. There were four conditions for which ERPs were extracted: those elicited by the episodic and semantic task-cues cues on switch and on repeat trials. On average, 84% of the available trials contributed to the ERP data for each participant. Mean trial numbers contributing to the ERPs (ranges in parentheses) were: episodic switch = 50 (31–56), episodic repeat = 34 (21–39), semantic switch = 49 (32–55), semantic repeat = 34 (23–40).

## 3 Results

### 3.1 Behavior

The likelihood of a correct inside/outside response at study was 97%. For the test data, Table 1 shows the probabilities of correct and incorrect old responses, as well as the conditional probabilities of correct location (inside/outside) judgments in the episodic task, and correct classifications in the semantic task. The data were collapsed across the inside/outside dimension. Preliminary analyses conducted on data separated by this dimension revealed no differences of note.

**Table 1:** Probabilities of correct and incorrect old judgments and location judgments in the episodic task and correct classifications in the semantic task, on switch and repeat trials. Probabilities for old words were calculated by collapsing across correct and incorrect location judgments. The ‘Location’ values are the conditional probabilities of a correct inside or outside judgment for words judged correctly to be old. Standard deviations are in parentheses.

	Switch	Repeat
*Episodic*		
Hit	0.89 (0.10)	0.88 (0.11)
False Alarm	0.21 (0.13)	0.16 (0.12)
Location	0.76 (0.13)	0.83 (0.12)

*Semantic*		
Correct Classification	0.75 (0.08)	0.74 (0.10)

The probability of a correct old response was calculated as the summed probability of old words attracting a correct or incorrect location judgment. Old/new discrimination scores (discrimination index: *Pr* = *p*(hit) – *p*(false alarm)) were reliably above zero for both trial-types (switch *Pr*: 0.68, *t*(31) = 26.80, *p* < 0.001, *d_z_* = 4.74, 99.9% CL; repeat *Pr*: 0.72, *t*(31) = 25.51, *p* < 0.001, *d_z_* = 4.51, 99.9% CL (Common Language effect size statistic, Lakens, 2013, McGraw and Wong, 1992) and a one-tailed planned *t* test (based on previous outcomes: Williams et al., 2016) revealed superior discrimination on repeat trials (*t*(31) = 1.89, *p* = 0.035, *d_z_* = 0.33, 63% CL). The probability of a false alarm was also higher on switch trials (*t*(31) = 2.71, *p* = 0.011, *dz* = 0.48, 68% CL).

The probabilities of correct location judgments were reliably above chance on switch (*t*(31) = 11.84, *p* < 0.001, *d_z_* = 2.09, 98% CL) and repeat trials (*t*(31) = 15.40, *p* < 0.001, *d_z_* = 2.72, 99.7% CL). Accuracy was superior on repeat trials (*t*(31) = 3.56, *p* = 0.001, *d_z_* = 0.63, 74% CL). For the semantic task, the probability of classifying the item according to the modal rating given by the original raters was not statistically different across trial-types (see Table 1).

A 2 (old/new) × 2 (trial-type) × 2 (task) ANOVA was conducted on the mean per-participant reaction times (RTs) for correct responses (see Table 2). As for the response accuracy data, these analyses were conducted collapsed across the inside/outside dimension. Reaction times were slower on switch trials (*F*(1, 31) = 16.81, *p* < 0.001, *d_z_* = 0.72, 77% CL). Main effects of old/new (*F*(1, 31) = 30.04, *p* < 0.001, *d_z_* = 0.97, 83% CL) and task (*F*(1, 31) = 15.12, *p* < 0.001, *d_z_* = 0.69, 75% CL) were moderated by an interaction between these factors (*F*(1, 31) = 8.57, *p* = 0.006, *η_p_^2^* = 0.22). In both tasks reaction times are slower for ‘correct when old’ than for ‘correct when new’ judgments, and the interaction reflects the fact that the magnitude of this change is smaller for the semantic task (∼100 ms) than for the episodic task (∼200 ms).

**Table 2:** Mean reaction times (ms) for correct responses on each task on switch and repeat trials. Standard deviations are in parentheses.

	Switch	Repeat
*Episodic task*		
Old	1521 (340)	1441 (357)
New	1312 (242)	1236 (232)

*Semantic task*		
Old	1573 (321)	1480 (275)
New	1500 (253)	1389 (232)

### 3.2 ERP analyses

**Fig. 2:**
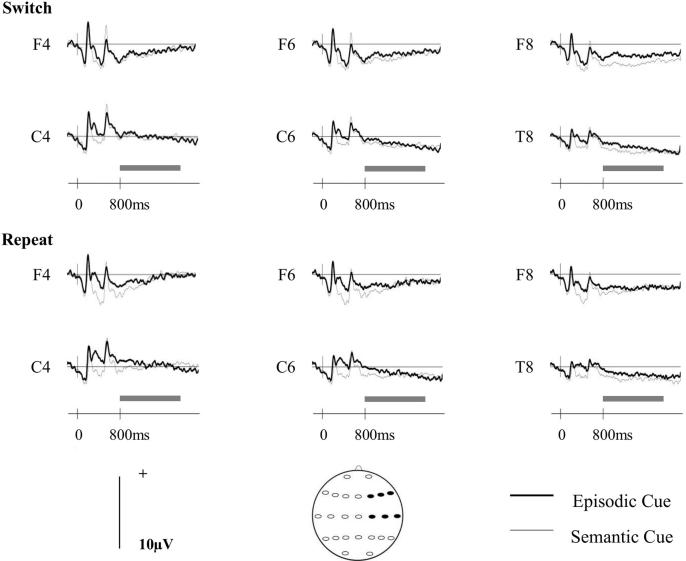
Grand average ERPs separated according to trial-type (switch/repeat) and cue-type (episodic/semantic) for right anterior (F4, F6, F8) and right central electrode sites (C4, C6, T8). The solid dark grey bars cover the 800–1900 ms time period.

**Fig. 3:**
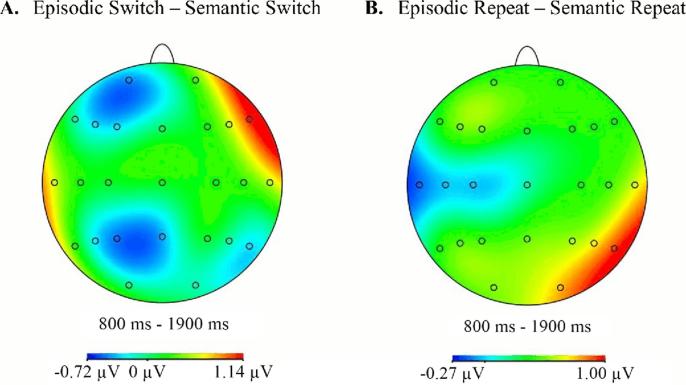
Topographic maps showing the differences between the scalp distributions of the neural activity associated with the episodic and semantic cues on switch (A) and repeat (B) trials from 800 to 1900 ms. The scale below each map denotes the voltage range (µV) of the differences between conditions.

Fig. 2 shows the grand-averaged ERP waveforms for each task-cue at right-frontal and central sites. The data are shown separately for switch and for repeat trials. Scalp maps depicting the differences between the scalp distributions of the ERPs associated with the different task-cues are shown in Fig. 3. These maps cover the 800–1900 ms period, during which low frequency changes linked to preparation for retrieval have been reported in previous studies (Evans et al., 2015, Herron and Wilding, 2004, Herron and Wilding, 2006a, Herron and Wilding, 2006b, Morcom and Rugg, 2002). Fig. 3 shows that there is a small relative positivity at right-frontal sites on switch trials for the episodic in comparison to the semantic task-cues, which is reversed to some extent over left-frontal sites. Fig. 3 also shows some posterior divergences according to task-cue on repeat trials.

The analysis strategy in this report followed that employed by Evans et al., 2015, Williams et al., 2016. The analyses were conducted on mean amplitudes taken over the 800–1900 ms post-stimulus time window. Following similar approaches in other studies (Herron and Wilding, 2004, Herron and Wilding, 2006a, Herron and Wilding, 2006b, Morcom and Rugg, 2002) the initial analysis included 12 sites distributed over left- and right-hemisphere frontal and central scalp (F3/F4, F5/F6, F7/F8, C3/C4, C5/C6, T7/T8) within an ANOVA incorporating the factors of task-cue (episodic/semantic), trial-type (switch/repeat), location in the anterior-posterior plane (anterior/central), hemisphere (left/right), and site (inferior/mid-lateral/superior). Only outcomes involving the factor of task-cue are reported.

The initial analysis revealed an interaction between task-cue, trial-type, anterior-central dimension, and hemisphere (*F*(1, 31) = 18.04, *p* < 0.001, *η_p_^2^* = 0.37). Separate ANOVAs were then carried out for switch and repeat trials. These revealed a task-cue by anterior-central by hemisphere interaction in both cases (switch: *F*(1,31) = 10.03, *p* = 0.003, *η_p_^2^* = 0.24; repeat: *F*(1,31) = 9.72, *p* = 0.004, *η_p_^2^* = 0. 24).

Follow up ANOVAs were subsequently carried out for the anterior and central sites separately. For switch trials differences were reliable only at the anterior sites, where there was an interaction between cue-type and hemisphere (*F*(1, 31) = 8.94, *p* = 0.005, *η_p_^2^* = 0.22). Fig. 3(A) shows that the largest divergence between the activities elicited by the two task-cue-types is over the right hemisphere, but separate ANOVAs for the two hemispheres revealed no reliable outcomes. The likely reason for the reliable interaction term is the relatively greater positivity for the episodic than the semantic condition at inferior right hemisphere sites, and a partial reversal of this over the left hemisphere.

For repeat trials differences were reliable only at central sites, where there was an interaction between task-cue-type and hemisphere (*F*(1,31) = 4.62, *p* = 0.04, *η_p_^2^* = 0. 13). Fig. 3(B) shows that the largest divergence comprises a greater relative negativity for neural activity elicited by episodic cues over left hemisphere central locations. Follow-up ANOVAs separately for each hemisphere revealed no reliable differences.

### 3.3 Bayesian ERP analyses

As noted in the Introduction, ERP activity that might reflect retrieval mode has been identified on switch trials in one recent study (Evans et al., 2015), and on repeat trials in several (for a comprehensive recent overview, see: Herron & Evans, 2018). The purpose of the Bayesian analyses reported here was to assess the strength of evidence separately for switch and repeat trials for the null hypothesis (no evidence of retrieval mode) or the alternative (Dienes, 2011, Verhagen and Wagenmakers, 2014). The motivation for these analyses was to provide a formal assessment of the correspondence between the findings here and those in previous studies in which reliable ERP divergences between comparable conditions over right-frontal scalp have been linked to retrieval mode. While the studies included are not identical in design, they have been selected because of their similarities, whilst also recognizing that the definition of mode entails that it should be engaged whenever episodic retrieval is required (Rugg and Wilding, 2000, Tulving, 1972). For each of the separate contrasts described below Bayes factors (BFs) of 3.0 and above were considered as substantial evidence for the alternative hypothesis, whereas BFs of 0.33 and below were considered substantial evidence for the null (Wasserman, 2004, Wetzels et al., 2011). BFs were calculated and plotted using the R-version of the Replication Test (Verhagen & Wagenmakers, 2014).

**Fig. 4:**
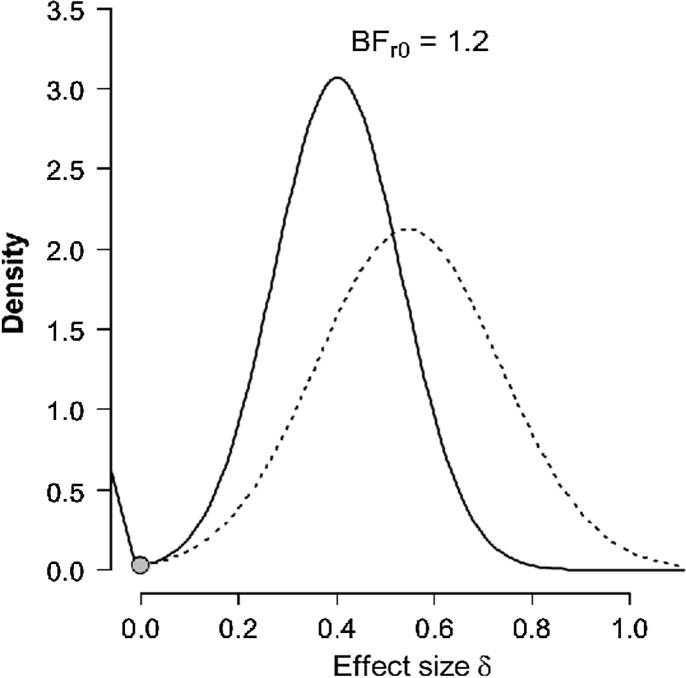
Bayesian result for the replication test of the right-frontal positivity identified previously during preparation for episodic memory retrieval on switch trials. The dotted line represents the posterior from the original study (Evans et al., 2015), which was used as the prior for the effect size in the replication test. The solid line represents the posterior distribution after the data from the replication attempt are taken into account. The grey dots indicate the ordinates of this prior and posterior for the null hypothesis that the effect size is zero. The ratio of these two ordinates gives the result of the replication test (Verhagen & Wagenmakers, 2014).

For switch trials, the outcomes in this experiment were set against those of Evans et al. (2015) where right-frontally distributed neural activity linked with retrieval mode was identified. The *t*-statistics were derived from the contrast between mean amplitudes elicited in response to the relevant task-cues. The mean amplitudes were the average taken from three right-frontal electrodes (F4, F6, F8) and in each case the time window of interest was 800–1900 ms after the presentation of the task-cues. When considered as a replication of the divergence obtained on switch trials in Evans et al. (2015) for this experiment the BF = 1.2 (Fig. 4). This is commonly interpreted as anecdotal evidence for the alternative hypothesis (Wasserman, 2004, Wetzels et al., 2011). Table 3 below provides a summary of *t*-values and sample sizes for each of the key experiments.

**Table 3:** *t* values and sample sizes (*N*) from previous studies demonstrating right-frontal positivity during preparation for episodic memory retrieval on switch/repeat trials, and for the replication attempt.

Study	*t* value	*N*
*Current experiment*		32
Analysis strategy as Herron and Wilding (2004)	1.06	
Analysis strategy as Herron and Wilding (2006a)	0.49	
Analysis strategy as Evans et al. (2015)	1.51	

Switch Contrast		
Evans et al. (2015)		32
Main effect of cue-type (location/perceptual)	3.09	

Repeat Contrasts		
Herron and Wilding (2004)		20
Main effect of cue-type (operation/semantic)	2.09	
Main effect of cue-type (location/semantic)	2.86	

Herron and Wilding (2006a)		16
Main effect of cue-type (location/semantic)	2.47	

**Fig. 5:**
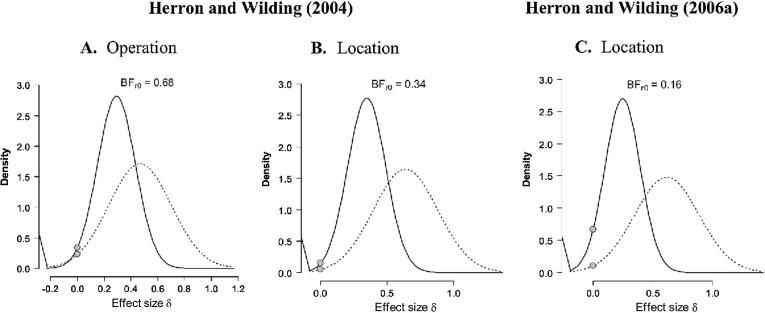
Bayesian results of the Replication Tests for the right-frontal positivity identified previously during preparation for episodic memory retrieval on repeat trials. Panels A and B are based on Herron and Wilding (2004). Panel C is based on Herron and Wilding (2006a). Other details as per legend for Fig. 4.

For repeat trials, the outcomes obtained here were set against those in two separate experiments in which location judgments were required in an episodic task condition. The first experiment is that reported by Herron and Wilding (2004). t values were calculated over the 800–1900 ms epoch using six right-frontal and central sites (F4, F6, F8, C4, C6, C8). In this experiment there were two episodic tasks: operation and location, and one baseline semantic task (see Table 3 below). As a replication of the operation task-cue effect in Herron and Wilding (2004) the BF = 0.68 (Fig. 5A). For a replication of the location effect the BF = 0.34 (Fig. 5B). These BFs indicate that the data provide moderate evidence in favor of the null hypothesis.

The second experiment for the assessment on repeat trials is that reported by Herron and Wilding (2006a). The relevant statistics for this output were computed over mean amplitudes for sites F4, F6 and F8, and the time period over which the data were averaged for Herron and Wilding (2006a) ran from 800 ms until 4000 ms after the task-cue, reflecting the extended epoch employed in that study. For a replication of the location effect in Herron and Wilding (2006a) (see Table 3) the BF = 0.16 (Fig. 5C). This indicates substantial evidence for the null hypothesis. In summary, across these different assessments there is no strong evidence that the right-frontal modulation linked to retrieval mode in prior work has been replicated. These outcomes are consistent with those reported earlier by Williams et al. (2016).

## 4 Discussion

This experiment was designed to develop further an understanding of the conditions under which ERPs are sensitive to indices of preparatory retrieval processing. The focus was on the process of retrieval mode, which Tulving defined as a cognitive state, entry into which ensures that subsequent events – such as test items on a memory task – modulate episodic retrieval processing (Tulving, 1983).

The empirical starting point for this investigation was null results obtained in two recent experiments (Williams et al., 2016). In those experiments there was little compelling evidence – via either frequentist or Bayesian analyses – for a particular ERP modulation that had been observed in several instances previously and linked to the process of retrieval mode. In this experiment, consideration of task-design elements that might have contributed to the null outcomes reported by Williams et al. (2016) prompted adoption of a design intended to provide a rigorous test of the possibility that right-frontal activity elicited by task-cues is an index of retrieval mode. The key departures from the designs of the two previous studies were as follows. First, the cue sequence was unpredictable. Second, a shorter RCI (500 ms vs 1200 ms) was employed. The reason for these changes, described in detail in the Introduction, was to maximize the conditions under which a domain-general index of preparation to retrieve episodic memories might be observed (Morcom & Rugg, 2002).

The frequentist analyses described above provide some evidence that the ERPs elicited by the task-cues differed. On repeat trials there was greater relative negativity for neural activity elicited by episodic cues over left hemisphere central locations. On switch trials ERPs elicited by episodic cues were somewhat more positive-going than those elicited by semantic cues at right-frontal electrode locations and more negative-going at left hemisphere sites. This outcome was reflected in a reliable interaction between condition and hemisphere. No reliable differences according to condition were evident when analyses were conducted separately for each hemisphere. While the general frontal asymmetry on switch trials is broadly similar to that reported previously, the modulation is small, and the rather focal divergence around F8 that can be seen in Fig. 3 is at odds with distributions reported elsewhere (cf. Evans et al., 2015, Herron and Evans, 2018, Herron and Wilding, 2004). Moreover, the outcomes of the Bayesian analysis for this effect on switch trials revealed no more than weak evidence for the view that the effect Evans et al. (2015) identified as signature of retrieval mode had been replicated.

How should these data be interpreted? In total, for four Bayesian analyses conducted over the data from three previously reported experiments, in each case restricted to electrode sites at which putative indices of retrieval mode have most commonly been observed, there was no strong evidence for the view that effects obtained previously were replicated. Moderate or strong evidence in favor of the null hypothesis was obtained in three of the four cases. In the earlier study by Williams et al. (2016) substantial evidence in favor of the null was obtained in both of the key analyses that were run. In so far as these outcomes might be seen to challenge the view that right-frontal activity elicited by task cues index retrieval mode, the data of Wilckens et al. (2011) are also relevant: they reported indices of preparation for retrieval that were largest at the vertex rather than over right-frontal scalp. Also of potential relevance here are the findings reported by Küper (2018) in an experiment where people prepared to make episodic or semantic memory judgments about studied pictures. ERPs elicited in response to episodic and semantic preparatory cues did not differ on switch trials. The design of the experiment, however, precluded a preparatory contrast on repeat trials. The consistencies in this set of outcomes contrast with the marked inconsistencies across these studies in the predictability of the sequences employed, as well as the RCIs. While it would be premature to rule out the possibility that these task demands have no influence on the magnitude of ERP modulations indexing preparatory retrieval processing, the available evidence suggests that they do not play a substantive role.

What other factors might explain the divergences across studies? Table 3 shows the sample sizes employed in this experiment and in the experiments against which replication assessments were carried out. These data provide little incentive to assume that this experiment is under-powered, and of course the sample size employed here was guided by the effect sizes reported in previous work. At the level of individual participants, other data relevant to signal:noise hence power considerations include the numbers of trials contributing to the averages for the response categories of interest. For switch trials, the mean number of trials entering into each of the episodic and baseline conditions was 50 in this experiment, 50 (exp1) and 51 (exp2) in Williams et al. (2016), and 54 in Evans et al. (2015). For repeat trials, means were 34 in this experiment, 51 (exp1) and 52 (exp2) in Williams et al. (2016), and 27 in Herron and Wilding (2006a). Herron and Wilding (2004) did not report mean trials numbers after artefact rejection. In their design the maximum number of trials before artefact rejection for all categories on switch and repeat trials is 40. These data in combination provide no compelling reason to take the view that the studies in which null results have been obtained are likely to have markedly inferior signal:noise to those in which reliable modulations over right-frontal scalp were revealed.

In a recent report Herron and Evans (2018) demonstrated that preparatory ERPs predict the accuracy of memory judgments (see also Evans & Herron, 2019), raising the possibility that differences between the ERPs in the critical categories in this experiment would be evident if the separation across tasks was restricted to trials for which the subsequent task judgment was correct. An important observation here is that this contrast was not reported in the previous experiments against which the outcomes described here have been compared. None the less, a related consideration is whether the levels of response accuracy in this experiment differ markedly from those in other studies. Differences of this kind would encourage consideration of the proportions of trials in average preparatory ERPs that might have supported accurate judgments, as well as the role played by task difficulty. Reassuringly, in two studies Herron and Wilding, 2004, Herron and Wilding, 2006a report very similar levels of response accuracy for both episodic and semantic retrieval tasks. In both of those experiments reliable differences between neural activities were revealed over right-frontal scalp when ERPs associated with preparation for episodic or semantic retrieval were contrasted. Thus, the presence/absence of differences between the critical classes of ERPs has been reported across studies where task difficulty and overall levels of accurate responding have been markedly similar.

In light of these considerations, perhaps the most straightforward way to explain the set of outcomes described above is to consider other demands across the episodic tasks in this experiment, the two earlier highly similar ones (Williams et al., 2016), and in the study by Wilckens et al. (2011). In the majority of the previous relevant studies the most common contrast has been between ERPs elicited by task-cues signaling semantic memory judgments and different kinds of episodic judgments: recognition memory (Düzel et al., 1999, Morcom and Rugg, 2002, Wilckens et al., 2011); location on screen at study (Evans et al., 2015, Herron and Wilding, 2004, Herron and Wilding, 2006a, Herron and Wilding, 2006b, Williams et al., 2016); the cognitive encoding operations people undertook at study (Herron and Evans, 2018, Herron and Wilding, 2004). While this list does not provide an obvious starting point for disentangling the task demands in the subset of studies under consideration here and those employed elsewhere, perhaps a more fruitful way in stems from the observation that pictures were employed at study and at test in this subset of experiments (Wilckens et al., 2011, Williams et al., 2016). In all of the other experiments referenced above, words were employed at study as well as at test.

This observation raises the possibility that preparatory retrieval processing for memory judgments is distinct when the memoranda are either words or pictures, and that the functional significance of an effect linked until now to retrieval mode should be re-visited. The data from studies in which pictures were employed cannot be used to argue in favor of the view that retrieval mode itself is not a real psychological construct, but they do prompt consideration of how the right-frontal activity reported on several occasions is best conceptualized. Rugg and Wilding, 2000, Wilding, 1999 introduced one conception of retrieval orientations, defining them as the preparatory processes that are engaged according to the specific demands that different episodic retrieval tasks impose. Neural signatures meeting the criteria for retrieval orientations have been reported in several studies (for review, see: Wilding & Ranganath, 2012), and a useful way forward may be to re-describe the functional significance of the right-frontal modulation in light of this definition. By this account, the right-frontal modulation is a retrieval orientation that is linked to preparation for retrieval of verbal contents. It is unlikely that this rather broad characterization is sufficiently granular, but it does provide an empirically sound starting point from which predictions can be tested in suitably designed experiments.

Also of relevance to these considerations is the observation that visual inspection of the scalp distributions of the effects that have been linked in previous studies to retrieval mode reveals some divergences in their maxima as well as the extent to which they are lateralized to the right hemisphere (Düzel et al., 1999, Herron and Evans, 2018, Herron and Wilding, 2004, Morcom and Rugg, 2002, Wilckens et al., 2011). While different scalp distributions are indicative of the engagement of different processes, some degree of variability across scalp distributions acquired via EEG is to be expected. This acknowledges the potential for differences that is introduced by recording from electrodes located on the scalp from caps with a fixed range of sizes. Moreover, the structural and functional heterogeneity of the frontal cortex is another relevant consideration (Ranganath and Knight, 2002, Stuss et al., 1994, Wheeler et al., 1995). This variability across previous studies might be subjected to a little more circumspection, however (at least with respect to considerations about retrieval mode), in light of the data in this paper and in the prior work where pictorial stimuli have been used in tasks where people prepare to make memory judgments.

Turning to the behavioral data in this experiment, reaction time (RT) switch costs were observed. This outcome is consistent with the view that the processes necessary for accurate memory judgments are challenged by the requirement to switch between tasks frequently. Herron and Wilding (2004) considered two (not mutually exclusive) possibilities for episodic switch costs. The first is that the volume or quality of task-relevant information that is recovered is lower on switch than on repeat trials. The second is that post-retrieval processes operating over recovered content are engaged later or less efficiently on switch trials. Both of these possibilities would increase the time it takes to make memory judgments on switch relative to repeat trials.

There are data points consistent with the first of these two accounts. First, ERP old/new effects shown to be sensitive to the volume of recovered episodic contents are smaller on switch than repeat trials (Evans et al., 2012, Wilckens et al., 2011). Second, ERP modulations that may signal a search for task-specific content are evident when people complete the same task over several trials, but not when frequent switches between tasks are required (Wilding & Nobre, 2001). These data suggest that one of the consequences of adopting retrieval mode is improved quality or volume of recovered memory contents.

The findings do not, of course, rule out the possibility that preparing to retrieve influences retrieval processing in other ways as well, and any combination of these might be employed to explain the changes in response accuracy that were also observed in this experiment. Old/new discrimination was superior on repeat trials, as was the case in the two previous experiments reported by Williams et al. (2016). In considering these outcomes, they noted the correspondence between the task demands here and those in experiments where the revelation effect has been reported (Watkins & Peynircioglu, 1990). In the revelation effect, people have to complete a task (for example ‘revealing’ the item to which a memory judgment is required by solving an anagram) before making memory judgments. Critically, the effect – which is commonly a decrement in accuracy on ‘reveal’ trials in comparison to trials where no ‘reveal’ is required - is also observed when different kinds of tasks are completed before the memory judgment is made (for example, solving an anagram for a different item; Frigo et al., 1999, Peynircioglu and Tekcan, 1993). Thus, to some extent, the comparison in revelation effect paradigms is similar to the switching requirements imposed here, raising the possibility that the processes responsible for these changes are the same (Williams et al., 2016). Moreover, in studies of the revelation effect changes in criterion are also commonly observed, and the difference in false alarm rates reported here is also suggestive of a correspondence between these phenomena (Azimian-Faridani and Wilding, 2004, Verde and Rotello, 2003, Verde and Rotello, 2004). In studies of the revelation effect the separate contributions that changes in sensitivity and changes in bias might contribute have been assessed successfully using receiver operating characteristics (ROCs; Verde & Rotello, 2004). Whilst presenting a challenge to implement, combining this approach with the manipulations employed here would go some way to disentangling how these two possible contributors are engaged on switch and on repeat trials.

The final element of the behavioral data reported here is the superior location memory on repeat trials. This outcome has been observed in some studies and not in others, and the effects are commonly small (cf: Evans et al., 2012, Herron and Wilding, 2004, Herron and Wilding, 2006b, Johnson and Rugg, 2006, Werkle-Bergner et al., 2005, Wilckens et al., 2011, Wilding and Nobre, 2001). These data do not permit strong conclusions to be drawn at this point.

In conclusion, the ERP data reported here, when considered alongside data obtained in similar experiments, challenge the functional account offered previously for sustained right-frontally distributed activity that has been observed while people prepare for episodic retrieval. This activity has been linked to the process of retrieval mode. A key criterion for a neural index of retrieval mode is that it is engaged whenever episodic retrieval is required. The absence of strong evidence for this modulation in this experiment and in other similar ones suggests that the functional significance of this neural signature could be amended. It may well be an index of a retrieval orientation.

## Funding

AW was supported by a PhD studentship from the School of Psychology at Cardiff University, and is currently supported by a grant from the Medical Research Council (MR/N01233X/1).
